# Monocytes, endoplasmic reticulum stress and metabolomics in dogs with multiple organ dysfunction syndrome treated by continuous venovenous hemodiafiltration

**DOI:** 10.18632/oncotarget.16533

**Published:** 2017-03-23

**Authors:** Yun-Peng Xu, Xiao-Lu Sui, Ai-Sha Zhang, Lei Ye, Feng-Juan Gu, Ji-Hong Chen

**Affiliations:** ^1^ Department of Nephropathy, The First Affiliated Hospital of Xinjiang Medical University, Urumqi, Xinjiang Uygur Autonomous Region of China, China

**Keywords:** multiple organ dysfunction syndrome (MODS), continuous venovenous hemodiafiltration (CVVHDF), monocytes, endoplasmic reticulum stress, metabolomics

## Abstract

**Objectives:**

We tried to investigate the mechanism of continuous venovenous hemodiafiltration (CVVHDF) treatment in monocytes function, endoplasmic reticulum (ER) stress signaling pathways, metabolomics and histopathological changes of MODS dogs, and aimed to enhance the understanding of pathogenesis and provide novel avenues to potential therapies.

**Methods:**

12 male Beagle dogs were used to develop the stable models of MODS by using hemorrhagic shock plus resuscitation and endotoxemia, and assigned randomly to CVVHDF group (n=6) and MODS group (n=6). The dogs in CVVHDF group were given the typical CVVHDF treatment for 24h after the completion of endotoxin intravenous infusion, while those in MODS group were offered the i.v heparin instead only. Serum sample were collected at five time points, i.e. before anesthesia, 0h, 6h, 12h and 24h after the endotoxin injection (T_1_˜T_5_, respectively), and meanwhile, the changes of mRNA, protein and human umbilical vein endothelial cells (HUVECs) apoptosis rates in JNK, CHOP and Caspase-12 were observed before and after interfered by RNA interference technology.

**Results:**

The levels of DLA-DR, IL-1β and IL-4 were higher than those in MODS group after the CVVHDF treatment, and the early and late apoptosis rates showed downward trend compared with MODS group. *In vitro* and prior to RNA interference (RNAi), the levels of mRNA and protein expression and HUVECs apoptosis rates of JNK, CHOP and Caspase-12 in CVVHDF group were significantly lower compared to T_1_ and MODS group respectively. However, the levels of mRNA and protein expression and HUVECs apoptosis rates were significantly lower than those before interfered by RNAi in both two groups. The serum levels of LPCs, ornithine, proline, methionine, etc. were down-regulated while carnitines, FFAs, PC, etc. were increased significantly in MODS (T_4_), and the serum levels of methionine, proline, arginine and lysine were increased while carnitine, LPCs, PCs, SMs and orthophosporic acid were decreased after 12 hours CVVHDF treatment (T4).

**Conclusion:**

CVVHDF treatment could reduce the apoptosis of the cells by enhancing the antigen presentation, improving the anti-inflammatory and proinflammatory imbalance and even correcting the metabolic disorder of amino acids and phospholipids.

## INTRODUCTION

Multiple organ dysfunction syndrome (MODS) is defined as dysfunction affecting two or more body organs or systems which occurs simultaneously or sequentially after shock, injury, infection, burn, etc [1]. The pathophysiology process of MODS is complicated, including a series of inflammatory responses, such as antigen presentation, antigen secretion, pro-inflammatory and anti-inflammatory responses [2, 3]. In recent years, in-depth study of endoplasmic reticulum (ER) stress and metabolic change provides a new perspective for the occurrence and development of MODS [4–7]. CVVHDF is a kind of purification method can effectively remove toxins and inflammatory substances of the blood, and it has been widely used in the treatment of MODS because it has the characteristics of high efficiency and safety, and the theory of endoplasmic reticulum stress has been gained acceptance on injury and sepsis.

Study has considered the imbalance in anti-inflammatory/pro-inflammatory cytokines, decline of monocyte antigen presentation function, ER stress and the disorder of amino acids and lipids which can stimulate or increase apoptosis of endothelial cells, further aggravating inflammation and dysfunction of various organs and creating a vicious cycle which can lead to MODS [5, 10–12]. ER stress results from a mismatch between ER protein load and ER protein folding capacity [13]. Its response is activated when cells experience various stressors, such as pyemia, ischemia/reperfusion(I/R), acidosis, hypoxia, low glucose levels, ATP depletion and alterations in calcium homeostasis [14, 15]. The ER stress response can promote either cell survival or cell death depending on the degree of injury and other factors [13, 16, 17].

As an important part of phospholipids of cell membranes *in vivo*, the formation of membrane lipid microenvironment controls the characteristics of many components. It also regulates the transport of substances *in vivo*, ligand binding to the receptor, cell differentiation and recognition, membrane signal reception and transmembrane signal transduction [18]. The ER stress in MODS induces apoptosis, which results in the change of membrane lipid microenvironment, and plasma phospholipid metabolism in the body also changes accordingly.

However, the specific mechanism of CVVHDF treatment and ER stress of MODS is still unclear. What's more, the amino acid and lipid mediators, especially lipid mediators, whose concentrations are usually limited in the body, are produced when necessary and promptly resolved according to inflammatory remission. That's also the reason why it is difficult to identify them. This paper aimed to explain the mechanism of CVVHDF in MODS from perspectives of monocytes function, ER stress apoptosis pathway and metabolomics.

## RESULTS

### Determination of monocyte function

**Figure 1:**
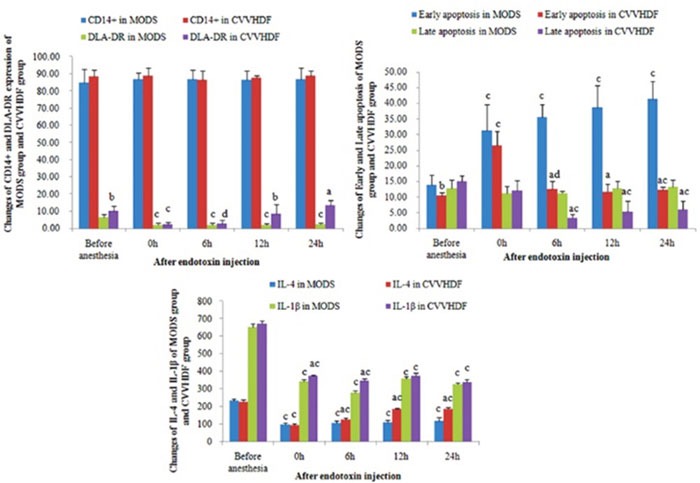
The level of CD14+, DLA-DR, early apoptosis, late apoptosis, IL-4 and IL-1β in MODS group and CVVHDF group (*^a^P <0.01,^b^P <0.05, CVVHDF group versus MODS group; ^c^P <0.01,^d^P <0.05, the indexes in the two group were compared with the indexes in their own group at T1 respectively*).

The mononuclear cells were isolated by the immunomagnetic beads from the mononuclear cell suspension and the average positive rate of CD_14_^+^ was all above 85% through the positive staining (Figure 1).

### Antigen-presenting function

The expression level of DLA-DR in CD_14_^+^ monocytes was low in normal conditions [9]. The experiment showed that the level decreased after the hemorrhagic shock and endotoxin double attack, and the DLA-DR was reduced to the minimum level when given the endotoxin 12h (T_4_). DLA-DR in CD_14_^+^ monocytes showed upward trend after the CVVHDF treatment, and its expression was higher than that in MODS group respectively at T_4_ and T_5_ (Figure 1, Table 1).

**Table 1:** Changes of CD_14_^+^, DLA-DR expression, apoptosis and secretion function of MODS group and CVVHDF group

Group	Project	T_1_	T_2_	T_3_	T_4_	T_5_
MODS	CD_14_^+^	85.08±7.75	87.12±3.40	87.08±5.07	86.72±5.32	87.02±6.67
	DLA-DR	6.52±1.47	2.15±0.94^c^	2.10±0.96^c^	1.77±1.01^c^	2.37±0.92^c^
	Early apoptosis	13.87±3.09	31.12±8.44^c^	35.58±3.85^c^	38.63±7.09^c^	41.32±5.61^c^
	Late apoptosis	12.72±2.78	11.13±2.40	11.15±0.75	12.75±2.30	13.28±2.22
	IL-4	232.59±9.60	98.45±7.15^c^	104.94±11.21^c^	107.92±13.06^c^	119.63±17.30^c^
	IL-1β	654.18±16.78	343.86±12.45^c^	278.18±11.66^c^	360.02±9.05^c^	328.32±7.16^c^
CVVHDF	CD_14_^+^	88.57±3.97	89.23±4.49	86.70±5.04	87.75±1.38	88.95±3.10
	DLA-DR	10.15±3.11^b^	2.28±1.42^c^	2.93±1.90^d^	8.45±5.60^b^	13.55±2.93^a^
	Early apoptosis	10.50±1.00^b^	26.45±4.55^c^	12.55±2.36^ad^	11.58±2.44^a^	12.40±0.75^ac^
	Late apoptosis	14.98±1.75	12.13±3.06	3.30±1.00^ac^	5.37±3.23^ac^	6.00±2.67^ac^
	IL-4	226.85±10.46	94.35±7.09^c^	126.85±6.86^ac^	184.28±5.97^ac^	184.42±7.87^ac^
	IL-1β	670.78±17.34	373.42±6.08^ac^	347.51±10.70^ac^	374.56±15.57^c^	339.95±16.36^c^

### Monocytes apoptosis

Apoptosis of CD_14_^+^ mononuclear cells in early and late stages increased gradually in the MODS group and began to decline gradually after the CVVHDF treatment (Figure 1 and Table 1).

### Monocytes secretion function

At the T1 point, the level of IL-1β and IL-4 which were secreted by monocytes was higher than that after the stimulation by endotoxin, while the monocytes had active secretory function in normal condition, and the secretion of IL-1β and IL-4 stimulated by endotoxin was decreased and maintained at a lower level in the MODS group (Figure 1 and Table 1).

### HUVECs apoptosis induced by endoplasmic reticulum stress

**Figure 2:**
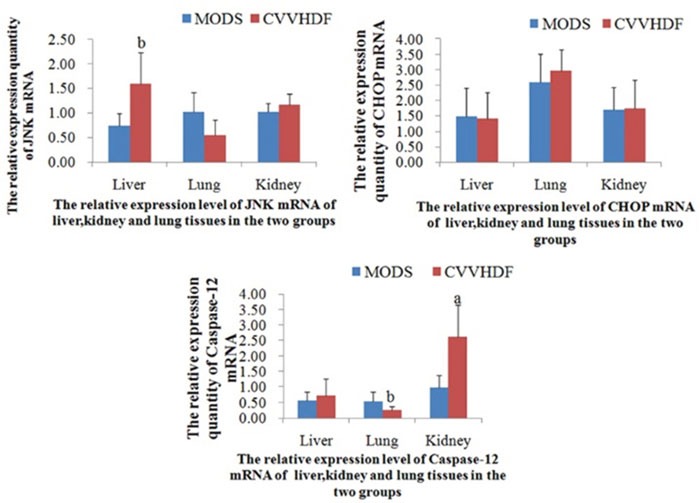
The mRNA relative expression of JNK, CHOP and Caspase-12 in Liver, Lung and Kidney tissues before interfered by siRNA The immunofluorescence quantitative PCR was used to detect the expression level of JNK, CHOP and Caspase-12 mRNA in liver, lung and kidney tissues. Values were expressed as mean ± SD, (*^a^P<0.01, ^b^P<0.05, CVVHDF versus MODS*).

*In vivo*, there was no significant difference between MODS and CVVHDF group in mRNA levels only except that JNK in liver, Caspase-12 in lung and kidney, and it was indicated that there was no significant difference generally (Figure 2 and Table 2).

**Table 2:** Gene expression levels of Caspase-12, CHOP and JNK2 in lung, liver and kidney (x¯±s)

Project	Group	Lung	Liver	Kidney
JNK2	MODS	1.01±0.40	0.74±0.24	1.01±0.18
	CVVHDF	0.54±0.31	1.59±0.64^b^	1.17±0.21
CHOP	MODS	2.60±0.91	1.47±0.93	1.69±0.73
	CVVHDF	2.97±0.68	1.40±0.87	1.75±0.90
Caspase-12	MODS	0.54±0.29	0.55±0.29	0.97±0.41
	CVVHDF	0.26±0.11^b^	0.73±0.53	2.63±1.03^a^

**Figure 3:**
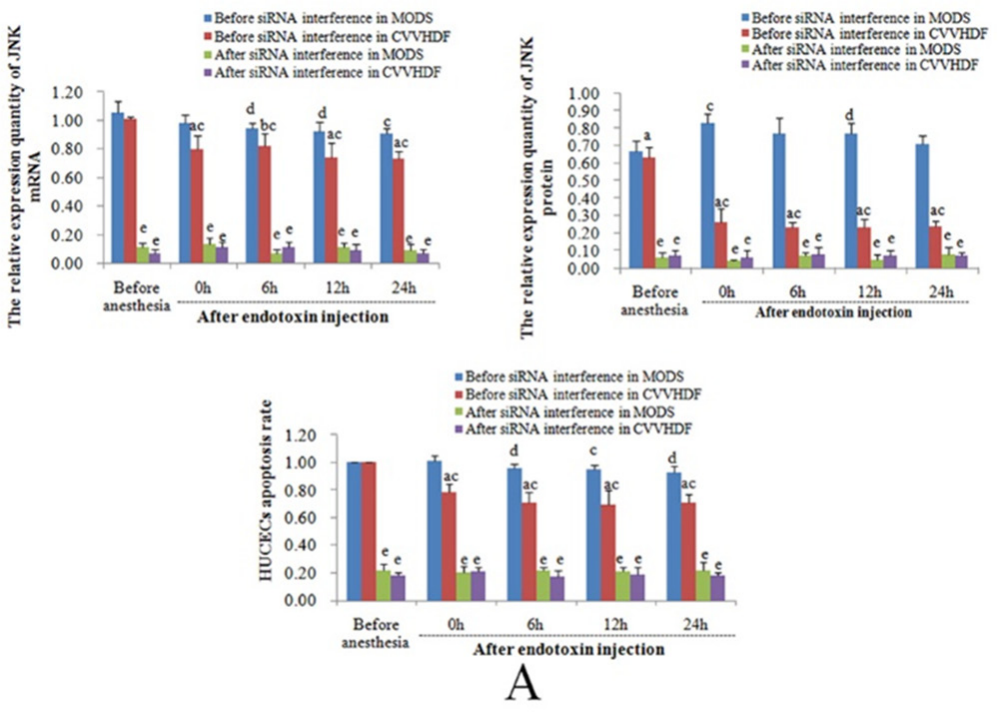
The levels of mRNA, protein and endothelial cell apoptosis among JNK, CHOP and Caspase-12 were compared between MODS group and CVVHDF group before and after interference **(A)** The relative expression level of JNK mRNA, protein and HUVECs apoptosis rates in MODS and CVVHDF group before and after interfered by JNK siRNA. **(B)** The relative expression level of CHOP mRNA, protein and HUVECs apoptosis rates before and after interfered by CHOP siRNA. **(C)** The relative expression level of Caspase-12 mRNA, protein and HUVECs apoptosis rates before and after interfered by Caspase-12 siRNA. (*^a^P<0.01, ^b^P<0.05, CVVHDF group versus MODS group before interfered; ^c^P<0.01, ^d^P<0.05, MODS group and CVVHDF group were compared with T1 in each group; ^e^P<0.01, ^f^P<0.05, the comparison of each group before and after interference at the same point*).

*In vitro* and prior to siRNA interference, the mRNA and protein expression and HUVECs apoptosis rates of JNK, CHOP and Caspase-12 at T_3_ to T_5_ in CVVHDF group were significantly lower compared to T_1_ and MODS group respectively, indicating that CVVHDF could alleviate the condition MODS effectively. After the siRNA-mediated Gene Silencing, the mRNA and protein expression of JNK, CHOP and Caspase-12 at T_1_ to T_5_ among the two groups was significantly lower than that before interference, and the HUVECs apoptosis rate was also maintained at a low level, indicating that the apoptosis of HUVECs was inhibited successfully after siRNA-mediated Gene Silencing (Figure 3 and Table 3).

**Table 3:** Comparison of JNK, CHOP and Caspase-12, mRNA, protein and endothelial cell apoptosis in the two groups before and after interference

Project	Category	Group	Interference	T1	T2	T3	T4	T5
JNK	mRNA	MODS	Before	1.05±0.08	0.98±0.06	0.94±0.04d	0.92±0.07c	0.91±0.03d
			After	0.11±0.03e	0.13±0.05e	0.07±0.03e	0.11±0.03e	0.09±0.04e
		CVVHDF	Before	1.01±0.01	0.80±0.09ac	0.82±0.09bc	0.74±0.10ac	0.73±0.05ac
			After	0.07±0.03e	0.11±0.04e	0.11±0.04e	0.09±0.04e	0.07±0.03e
	Proteins	MODS	Before	0.67±0.06	0.83±0.05c	0.77±0.09	0.77±0.06	0.71±0.05
			After	0.06±0.03e	0.04±0.01e	0.07±0.02e	0.05±0.03e	0.08±0.04e
		CVVHDF	Before	0.63±0.06	0.26±0.08ac	0.23±0.03ac	0.23±0.05ac	0.24±0.03ac
			After	0.07±0.03e	0.06±0.04e	0.08±0.04e	0.07±0.03e	0.07±0.02e
	Apoptosis	MODS	Before	1.00±0.00	1.01±0.04	0.96±0.03d	0.95±0.03c	0.93±0.04d
			After	0.22±0.04e	0.20±0.05e	0.22±0.02e	0.21±0.03e	0.22±0.06e
		CVVHDF	Before	1.00±0.00	0.78±0.06ac	0.71±0.07ac	0.69±0.11ac	0.71±0.06ac
			After	0.18±0.02e	0.21±0.03e	0.17±0.05e	0.19±0.05e	0.18±0.02e
CHOP	mRNA	MODS	Before	0.91±0.03	0.89±0.08	0.92±0.03	0.84±0.08	0.88±0.07
			After	0.11±0.04e	0.10±0.02e	0.09±0.03e	0.13±0.03e	0.12±0.02e
		CVVHDF	Before	0.96±0.15	0.89±0.05	0.69±0.11ad	0.64±0.07ac	0.50±0.11ac
			After	0.10±0.03e	0.12±0.03e	0.11±0.05e	0.11±0.03e	0.11±0.05e
	Proteins	MODS	Before	0.81±0.05	0.80±0.05	0.80±0.05	0.82±0.07	0.82±0.03
			After	0.11±0.02e	0.11±0.03e	0.13±0.02e	0.13±0.04e	0.11±0.03e
		CVVHDF	Before	0.82±0.04	0.27±0.03ac	0.22±0.04ac	0.29±0.03ac	0.20±0.04ac
			After	0.10±0.02e	0.15±0.04e	0.17±0.02e	0.14±0.02e	0.10±0.03e
	Apoptosis	MODS	Before	1.00±0.00	1.01±0.04	0.96±0.03d	0.95±0.03c	0.93±0.04d
			After	0.29±0.05e	0.29±0.03e	0.26±0.04e	0.27±0.03e	0.21±0.04e
		CVVHDF	Before	1.00±0.00	0.78±0.06ac	0.71±0.07ac	0.69±0.11ac	0.71±0.06ac
			After	0.25±0.06e	0.24±0.10e	0.24±0.03e	0.21±0.07e	0.21±0.03e
Caspase-12	mRNA	MODS	Before	1.01±0.09	0.94±0.04	0.93±0.05	0.94±0.05	0.89±0.06c
			After	0.57±0.04e	0.19±0.07e	0.42±0.08e	0.49±0.06e	0.42±0.08e
		CVVHDF	Before	0.96±0.05	0.82±0.03ac	0.70±0.04ac	0.77±0.05ac	0.69±0.10ac
			After	0.35±0.07e	0.23±0.08e	0.29±0.05e	0.25±0.07e	0.31±0.07e
	Proteins	MODS	Before	0.03±0.02	0.41±0.05c	0.36±0.04c	0.34±0.05c	0.37±0.02c
			After	0.21±0.04e	0.16±0.04e	0.15±0.03e	0.12±0.03e	0.05±0.02e
		CVVHDF	Before	0.06±0.03	0.12±0.05ad	0.14±0.03ac	0.15±0.03ac	0.13±0.03ac
			After	0.23±0.07e	0.10±0.03	0.13±0.02	0.11±0.04	0.06±0.03e
	Apoptosis	MODS	Before	1.00±0.00	1.01±0.04	0.96±0.03d	0.95±0.03c	0.93±0.04d
			After	0.68±0.04e	0.48±0.02e	0.44±0.04e	0.44±0.09e	0.36±0.11e
		CVVHDF	Before	1.00±0.00	0.78±0.06ac	0.71±0.07ac	0.69±0.11ac	0.71±0.06ac
			After	0.70±0.04e	0.51±0.07e	0.36±0.16e	0.33±0.07e	0.33±0.06e

### Serum metabolomics analysis

**Figure 4:**
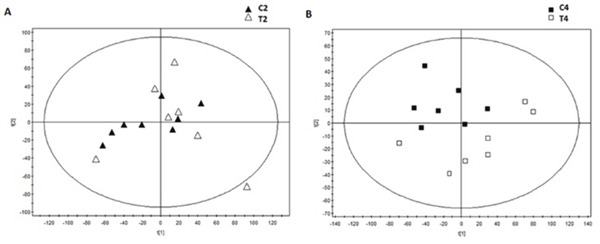
The score scatter plots of PCA based on metabolite contents of the MODS group and CVVHDF group at T2 point (A) and T4 point (B)

The serum was analyzed by UPLC Triple Q TOF MS. Meanwhile, a specific standard for the intensity of metabolite ions had been established, guaranteeing a minimum of RSD. Finally, the test showed that the percent of RSD which was less than 30% was reached in 94.6% among the 5410 ions in positive models, and the RSD less than 30% was reached in 78.6% among the 8180 ions in negative models. It was demonstrated that it's good for the next metabolomics study. Meanwhile, it was also found that MODS group and CVVHDF group were not separated at T_2_ by using principal component analysis (PCA) (Figure 4A), while separated significantly at T_4_ (Figure 4B), indicating that there was no significant difference in metabolic profiling between two groups before CVVHDF treatment while showing significant difference at T_4_. Thus, the T_4_ was selected to investigate metabolic changes after treatment by CVVHDF.

**Figure 5:**
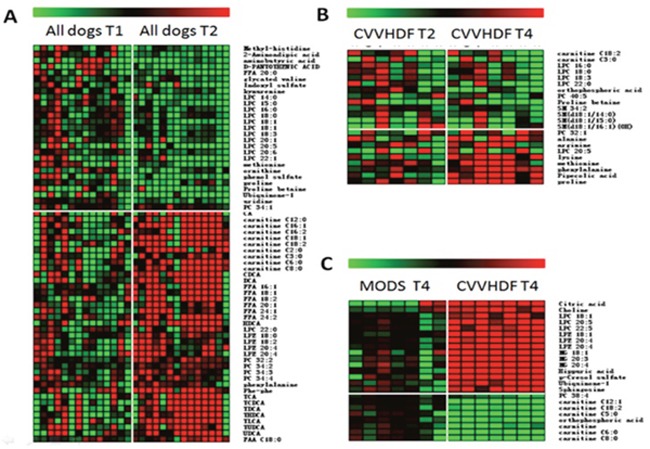
Heat maps of differential metabolites (A) Comparison of T2 and T1; (B) Comparison of T4 and T2 in the CVVHDF group; (C) Comparison of the MODS group and CVVHDF group at T4 point

The metabolites were analyzed by MS/MS ion fragment pattern and some differential metabolites had been found between T_1_ and T_2_ in all dogs. LPCs, ornithine, proline and methionine were down-regulated compared with the T_1_, while carnitines, FFAs, PC and bile acids increased significantly (Figure 5A). After 12-hour CVVHDF treatment (T_4_), the abnormal serum levels of the metabolic group began to return to normal levels, i.e methionine, proline, arginine and lysine were increased, while the serum levels of carnitine, LPCs, PCs, SMs and orthophosphoric acid were decreased (Figure 5B). The multiple comparisons had been used to show the further difference of metabolomics data between CVVHDF group and MODS group at T_4_. In T_4_, the p-cresol sulfate, citric acid, choline, hippuric acid, LPCs, LPEs and MGs, etc. increased in CVVHDF group compared with MODS group. In addition, carnitine and PC38:4 were shown to decrease in CVVHDF group (Figure 5C).

### Histological analysis

**Figure 6:**
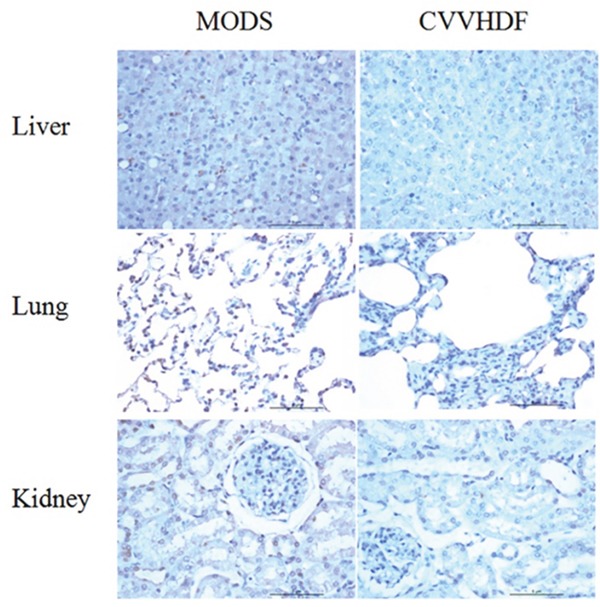
Pathological change of Liver, Lung and kidney in MODS group and CVVHDF group

In MODS group, narrowing alveolar septum and fracture adjacent alveoli were integrated into a larger cavity; alveolar epithelia, the capillary dilatation and congestion were associated with alveolar hemorrhage (Figure 6). Liver normal liver plate structure disappeared, with fatty degeneration of the liver cell atrophy. There were spotty necrosis, sinusoidal expansion bleeding, and periportal bile duct proliferation accompanied by inflammatory cell infiltration. Compared with MODS group, the lung pathology of CVVHDF group was characterized by mitigate damage, less alveolar wall rupture, less alveolar integration and hemorrhage, otherwise. An increase in the number of glomeruli, part of the balloon wall thickening, tubular swelling, and bleeding were associated with renal interstitial infiltration of inflammatory cells.

## DISCUSSION

In this study, we explored the mechanism of CVVHDF in the treatment of MODS in terms of immunity, amino acid and lipoids metabolism, and analyzed its effect on the improvement of histology. In addition, our data revealed the mechanism of RNA interference technology to reduce the apoptosis in terms of JNK, CHOP and Caspase-12 signal pathways in endoplasmic reticulum stress apoptosis models for the first time. It was demonstrated that CVVHDF treatment improved the imbalance of pro-inflammatory/anti-inflammatory cytokines, histological damage and disorder of metabolomics, including amino acids and lipids, and siRNA interference (siRNA-mediated Gene Silencing) on JNK, CHOP and Caspase-12 also reduced the HUVECs apoptosis rate.

The function of mononuclear cells can reflect the immune function of the body. Our results showed that mononuclear cells of early and late apoptotic rates in CVVHDF group had decreased significantly compared with the MODS group, and our previous study results showed that the mononuclear cells of peripheral blood were significantly increased after the treatment by CVVHDF about the “two-hit” MODS models. It was indicated that CVVHDF could significantly reduce the apoptosis of monocytes, and enhance the body's immunity to external stimuli. The expression of monocyte HLA-DR (DLA-DR in dogs) plays a vital role in the initiation of lymphocyte antigen presentation and immune response. In MODS course, the low expression levels of CD_14_^+^ can suppress the normal immune massive response which can cause cell damage and MODS [9].

In recently years, studies have shown that the reduction of HLA-DR antigen expression in the course of MODS may be related to the following factors: 1) The body's stress response and systemic inflammatory response syndrome (SIRS) stimulated the release of inflammatory mediators, and excessive anti-inflammatory reaction reduced the expression of HLA-DR antigen *in vivo* [19, 20]. 2) The body released a large amount of endotoxin, which could inhibit the ability of γ-IFN stimulating the expression of HLA-DR antigen, thereby decreasing the expression of HLA-DR [21]. 3) Mononuclear cells in peripheral tissues or their HLA-DR antigen synthesis function were consumed too much. In our experiment, the expression level of DLA-DR was higher than that of T_1_ time and MODS group, and CVVHDF helped to maintain a stable internal environment. Then the presentation function was improved and MODS was alleviated.

IL-1β is a pro inflammatory mediator secreted by macrophages and monocytes in the early stage of inflammation [22, 23], while IL-4 belongs to an anti-inflammatory mediator secreted by Th2 cells, mast cells and eosinophils [24]. They both maintain the balance between pro-inflammation and anti-inflammation *in vivo*. The single nucleus cells have active secretory function under normal conditions, and IL-1 beta and IL-4 are in high level *in vivo*. In the pathologic process of MODS, IL-1β and IL-4 which were released by monocytes were stimulated by endotoxin and maintained a low level, with the ongoing CVVHDF treatment, IL-4 secretion was increased significantly but did not reach the level before the experiment while IL-1β maintained a low level. It was demonstrated that MODS may partially inhibit the reaction of immune cells, while CVVHDF may clean the inhibition substance of IL-4.

ER stress is a cellular self-protection mechanism, which induces unfolded protein response (UPR) to protect cells from damage [25]. Moderate ER stress helps restore cell function, while the excess inflammatory reaction leads to the accumulation and damage of the wrong folding proteins which can cause severe cell apoptosis [26], which is caused by activating the proapoptotic transcription factor CCAAT/enhancer binding protein (C/EBP) homologous protein (CHOP), or JNK, or caspase-12.

**Figure 7:**
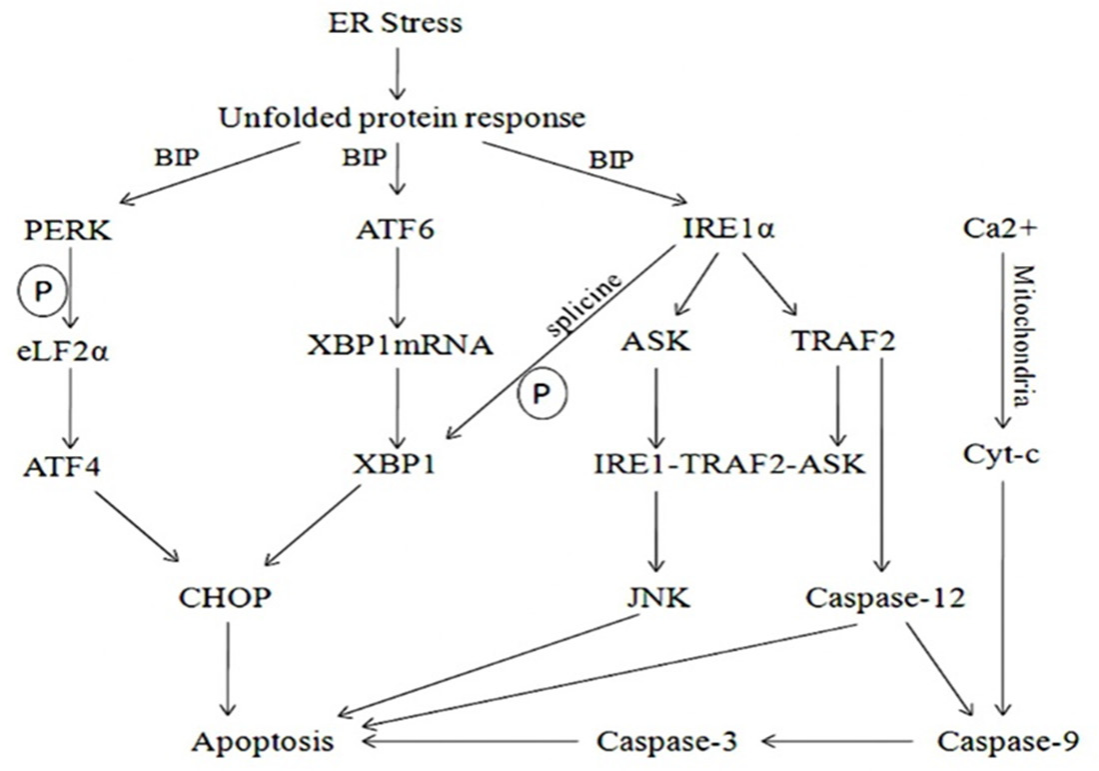
Upon ER stress, sequestration of BiP by unfolded proteins activated PERK, ATF6 and IRE1 Activation of the CHOP gene was induced by the activated PEPK and ATF6 pathways in the CHOP transcriptional activation pathway. Activated PERK mediated phosphorylation of eIF2α, resulting in repression of general translation, but permitting ATF4 translation, eventually leading to transcriptional upregulation of multiple genes including CHOP. The cleavage of ATF6 from the Golgi membrane facilitated its localization to the nucleus. Activated IRE1α was implicated into several functions: One of these functions was essential to drive the splice mechanism of the XBP-1 mRNA to allow the translation of mature XBP-1 protein that, in turn, functioned as a transcription factor to promote the transcription of UPR target genes such as CHOP leading to apoptosis; the other function of the activated IRE1α was to recruit TRAF2 and ASK that subsequently mediated the phosphorylation of ASK1 and formed the IRE1/TRAF2/ASK1. Thus apoptosis was induced through activation of JNK. The calcium exchange between ER and the cytosol eventually leaded to cytochrome c release and the onset of apoptosis. The mitochondria-dependent and -independent apoptotic pathways converged at caspase-9 and Caspase-3.

*In vivo*, the ER stress mainly induces apoptosis through the activation of CHOP transcription, activation of JNK and caspase-12 activation of three pathways [27]. Activation of the CHOP gene is induced by the activated PEPK and ATF6 pathways in the CHOP transcriptional activation pathway [28, 29], and the activation of CHOP is involved in the regulation of the expression of the downstream apoptosis related genes; activation of the IRE1 pathway in the JNK can interact with TRAF2 to raise the apoptotic signal regulated kinase (ASK1) to form the IRE1/TRAF2/ASK1 [30], thus inducing apoptosis through activation of JNK; Caspase-12 is involved in the activation of downstream Caspase-9 and Caspase-3 which lead to apoptosis without the participation of cytochrome C [31, 32] (Figure 7). *In vitro* and prior to siRNA interference, in our study, the mRNA and protein expression and HUVECs apoptosis rates of JNK, CHOP and Caspase-12 in CVVHDF group were significantly lower compared to T_1_ at T_3_ to T_5_ and MODS group respectively. It was revealed that CVVHDF could reduce the JNK, chop, caspase-12 expression in tissues effectively. In addition, we used two pairs of specific siRNAs to knock down the expression of JNK, CHOP and Caspase-12 in the HUVECs cells. As shown in the Table 3, the silencing of JNK, CHOP and Caspase-12 by siRNA significantly reduced the mRNA and protein expression and endotoxin-induced apoptosis rates both in MODS and CVVHDF group. siRNA interference could decrease JNK, CHOP, Caspase-12 expression, and ultimately relieve the MODS progress through interfering with the three pathway related gene sites.

FFAs can be used as energy supply to produce ATP through β-oxidization using carnitines as the transport into the mitochondria [33]. When the MODS model was established (T_2_), serum free fatty acids and carnitine were significantly increased. However, the level of carnitine in the CVVHDF group decreased significantly after 12-hour CVVHDF treatment, and meanwhile, we obtained the similar results compared with MODS group, suggesting that the β-oxidization in MODS models was reduced effectively after CVVHDF treatment. Our results also showed that the level of citric acid in CVVHDF group was significantly higher than that in the MODS group at T4 point. Citric acid is an important component of Tricarboxylic acid cycle (TCA, or Kreb's cycle) which is the main way to produce ATP. As one of the most important energy sources in the body, ATP has provided sufficient energy for metabolism *in vivo*. Previous studies showed that the TCA and electron transfer process were enhanced in the course of injury or sepsis, which can increase the energy supply to some extent, while the excessive oxidation can lead to further damage [34, 35]. The TCA occurs mainly in the mitochondria and citric acid content is increased, strengthening the role of TCA. It is thus indicated that CVVHDF treatment could also improve mitochondrial function.

**Figure 8:**
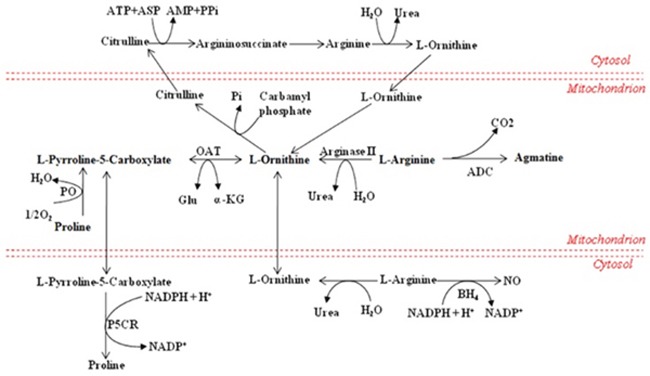
Metabolism of amino acids Partial amino acid metabolism in mammals. *ADC* arginine decarboxylase, *Asp* aspartate, *BH4* (6R)-5,6,7,8-tetrahydro-L-biopterin, *OAT* ornithine aminotransferase, *PO* proline oxidase, *P5CR* pyrroline-5-carboxylate reductase.

**Figure 9:**
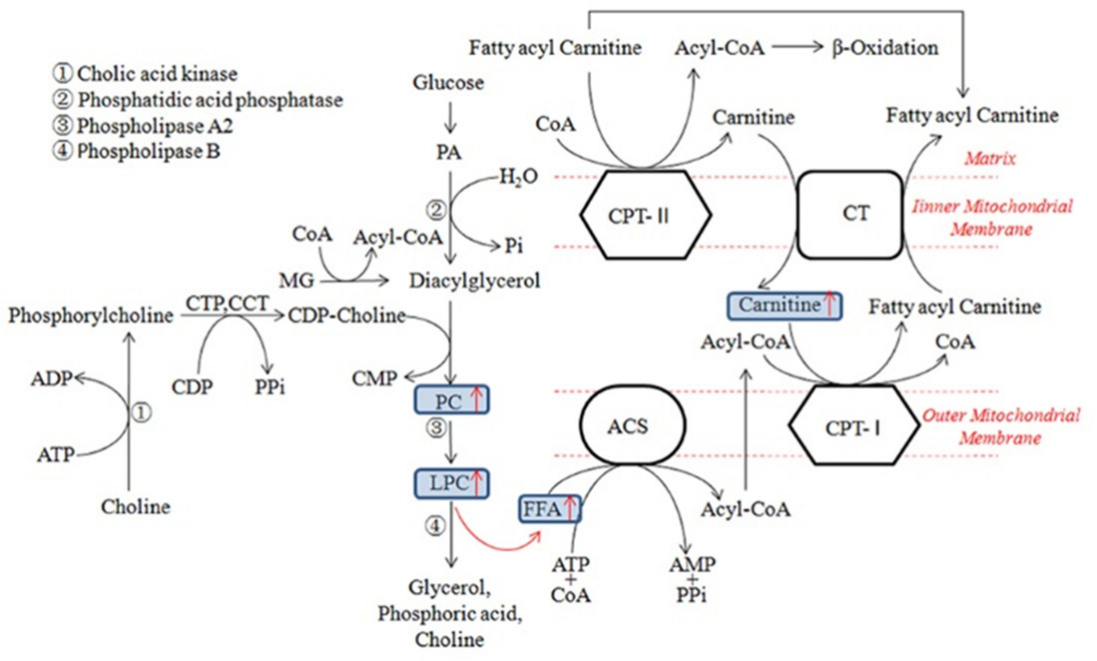
Mechanistic models of phospholipids metabolic alterations in the progress of MODS Oxidative stress or other external stimuli activated the metabolism of PC and caused the up-regulation of LPC. LPC in the progress of inflammation indicated the up-regulation of FFA. Similarly, increased FFA levels were observed in the MODS models which were positively correlated with carnitine levels. *(ACS Acyl-CoA synthetase, CPT-I Carnitine acyl transferase I, CPT-II Carnitine acyl transferase II, CT Carnitine-acyl carnitine translocase, FFA Free atty acid, PA Phosphatidic acid, PC Phosphatidylcholine, LPC Lysophosphatidyl choline, MG Monoacylglycerol, CCT phosphoryl choline cytidylyl transferase).*

The research showed that methionine was significantly increased in demand in trauma and infection conditions [36], which could also increase the production of reactive oxygen species and the clearance rate of free radical in mitochondria [37]. Similarly, the overall augmentation of methionine in the CVVHDF group supported the beneficial effect of CVVHDF treatment to achieve a better response to the anti-inflammatory effect. The intestinal absorption of arginine occurs via a transport system which shares with lysine, ornithine, and cysteine, and plays a momentous role in improving the inflammatory reaction and immune response [7, 38] (Figure 8), especially in cardiovascular, lung, kidney, gastrointestinal and liver immune regulation [39, 40]. The low levels of methionine, arginine and proline could be rectified significantly after CVVHDF treatment by comparing two groups. What's more, methionine and lysine could also clear free radical and improve immune function *in vivo* [37]. LPCs, also called lysolecithins, which are known to be highly bioactive molecules that can signal through G-protein–coupled receptors, play an vital role in the process of inflammation and cell proliferation (Figure 9). LPC has also been identified as a ligand for the immunoregulatory receptor G2A, which is predominantly expressed in immature T-cells and B-cells [41]. In the present study, LPCs were down-regulated in MODS group, but the level of LPCs was significantly higher in CVVHDF group at T4. Some research also showed that excessive immune response could deplete LPCs. Thus, we consider the decreased LPCs might be related with excessive immune response *in vivo*, while CVVHDF could reduce the excessive consumption of LPCs.

Sphingomyelins (SMs) are involved in the composition of cell membranes, which play a significant role in promoting lipid transport and apoptosis [42, 43]. The serum level of SMs was down-regulated after CVVHDF treatment for 12 hours (T_4_), and we assumed that the early and direct alterations of lipid rafts might induce oxidative stress which could lead to the MODS, while CVVHDF could maintain the balance of lipid metabolism by improving the imbalance of SMs. Thus the MODS state could be alleviated. Otherwise, tissue injuries in liver, lung and kidney were improved and the inflammation was reduced under the light microscope in CVVHDF group compared with MODS group, respectively.

In summary, the present study provides *in vivo* evidence that CVVHDF can remove the inflammatory mediators, reduce the apoptosis of endothelial cells, regulate immune homeostasis, and improve the disorder of amino acids and lipids. In addition, CVVHDF therapy enhances the antigen presentation and improves the imbalance between pro-inflammation and anti-inflammation. The noteworthy is that endoplasmic reticulum stress signaling pathway plays a significant role in the evolution of MODS, and the substantial effects of siRNA interference on signaling pathways suggest the potential of broad application in the treatment of MODS, which may be a new therapeutic target for the treatment of MODS in the future.

## MATERIALS AND METHODS

### Animal models

A total of 12 male Beagle dogs (15±2kg by weight) were provided by the Laboratory Animal Center (Xinjiang Medical University, China). with approval by the ethics committee on animal research. All the dogs were used to develop the stable models of MODS by using hemorrhagic shock plus resuscitation and endotoxemia that would imitate clinical features. The mean arterial pressure (MAP) was maintained 6.0-7.3kPa during the hemorrhagic shock, and then the lost blood and the amount of Ringer's solution equal to two times of the blood loss were rapidly intravenously dripped through the jugular vein. Twelve hours later, the endotoxin (Escherichia coli serotype 0111:B4, Sigma, USA) was intravenously injected at 1.5 mg/kg for 12h.

### Grouping and sample collection

The dogs were assigned randomly to CVVHDF treatment group (n=6) and MODS group (no treatment, n=6). The dogs in CVVHDF group were given the typical CVVHDF treatment for 24h after the completion of the endotoxin intravenous infusion, while those in MODS group received the i.v heparin at the same dosage as CVVHDF group instead. Serum sample were collected at five time points, i.e. before anesthesia, 0h, 6h, 12h, 24h after the endotoxin injection (T_1_˜T_5_, respectively).

### Continuous venovenous hemodiafiltration

The vascular access of CVVHDF was established by the right internal jugular vein (or femoral vein). Intelligent bedside renal replacement therapy machines (PRISMA-Flex, Hospal, Sweden) with a membrane area of 0.9m^2^ and the molecules weighting from 20Da to 30000Da filtered were used during the whole experiment. The blood flow was set at 150ml/L, and the flow rate of the dialysis, displacement liquid (bicarbonate at 3.35mmol/L, calcium at 2.3mmol/L, sodium at 143.6mmol/L, potassium at 3.79mmol/L, chloride at 116.7mmol/L, magnesium at 1.57mmol/L and glucose at 6mmol/L) and ultrafiltration were set at 1000ml/h, 1000ml/h and 2000ml/h, respectively. All the liquids were deployed before use to avoid the precipitation of calcium with carbonate. By using the pre filter heparin anticoagulation method, 5000U/L heparin saline 1.5L and heparin were intravenously dripped in a dose of 1000 IU/kg in systemic anticoagulation in the first experiment, followed by 1000 IU/h. The ultrafiltration volume was established by determination of the volume needed for treatment and physiological requirements.

### Preparation of monocytes

Peripheral blood which contained 0.2mmol/L of ethylenediaminetetraacetic acid disodium was suspended in phosphate buffered saline (PBS) and put on lymphocyte separation liquid, making the ratio of cells in suspension and separation volume 2:1, which underwent centrifugation at 2000r/min for 30 minutes. The membranoid individual nucleus cells were aspirated by a glass straw and washed by PBS for 3 times, then suspended in the serum containing EDTA and incubated for 20min. Finally, they were incubated in RPMI-164 (Gibco, USA) for 20min once more after washed twice in PBS. The non-adherent lymphocytes were removed by Hank solution. In addition, the single-cell suspension was washed 3 times in Hank solution, and then mononuclear cell suspension was harvested.

### Detection of monocyte antigen presentation, apoptosis and secretion function

Flow cytometry was used to detect the positive rate of DLA-DR CD_14_^+^ cells. The mononuclear cells were isolated by the immunomagnetic beads from the mononuclear cell suspension, and Annexin V assay was used to measure the cell apoptosis [8]. Interleukin (IL)-4 and IL-1β were selected as the detection index of the secretion function of monocytes and measured in supernatant by the enzyme-linked immunosorbent assay (R&D, USA).

### The expression levels of JNK, CHOP and Caspase-12 in liver, lung and kidney *in vivo*

The liver, lung and kidney tissues were collected, frozen and stored at -80°C until assay when the dogs were killed. The mRNA expression levels of JNK, CHOP and Caspase-12 in liver, lung and kidney were quantified by real time fluorescent quantitative PCR (ABI7500 Fast, Sangon, China).

### Construction of the human siRNA lentivirus vector and stable cell line

**Figure 10:**
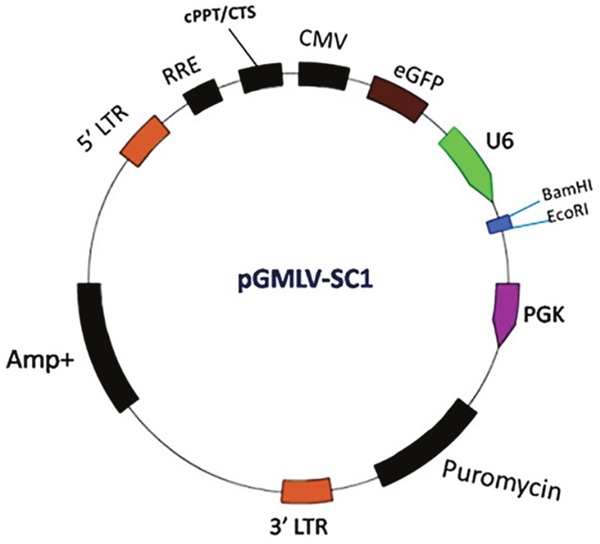
The map of Pgmlv-SC1 RNAi lentivirus vector

The Pgmlv-SC1 RNAi lentivirus vector is shown in Figure 10, and the four lentivirus siRNAs of JNK, CHOP and Caspase-12 gene were created based in the Pgmlv-SC1 RNAi lentivirus vector, respectively. The target spots are shown in Table 4, and HUVECs of target cells were infected by packed lentivirus and lentiviral negative controls. Western blot and Real Time PCR had verified the interference effect and selected the best silent lentivirus siRNA as the target cells, respectively (The siRNA4 in JNK, the siRNA1 in CHOP and Caspase-12), and then the stable strains were harvested in JNK, CHOP and Caspase-12.

**Table 4:** The target spots of siRNA in JNK, CHOP, and Caspase-12 gene

Gene name	NO.	TargetSeq	%GC
**JNK**	siRNA1	GGAGAGCTGGTGAAAGGTTGT	52%
	siRNA2	GCATTCAGCTGGTATAATTCA	38%
	siRNA3	GGATAAATGTTGCAGTCAAGA	38%
	siRNA4	GATGTGTATTTGGTTATGGAA	33%
**CHOP**	siRNA1	GGGACAGCCCATGATTAATTT	43%
	siRNA2	GGTGGAGGTGGAGGTAACTAT	52%
	siRNA3	GCTTGAGGTCATTGACATTAT	38%
	siRNA4	CGGACTATGTAATTGTAACTA	33%
**Caspase-12**	siRNA1	GCAAGGTAACATCTGTAATGA	38%
	siRNA2	GCCAGAGTCTGAAAGACAAAC	48%
	siRNA3	GGATCAAGAGCCAGATGTTCT	48%
	siRNA4	GCACATTCCTGGTGTTTATGT	43%

### Construction of the HUVECs and endoplasmic reticulum stress-induced apoptosis models

The HUVECs (Gibco, USA) after resuscitation were routinely placed in DMEM under the condition of 37°C, 5% CO_2_ and saturated humidity for 24 hours. Serum-free culture medium was added to the dog cerum in which the concentration reached 10% in each group at each time point, and the heparin anticoagulation serum 15ml was reserved at each time point respectively. HUVECs cultured endothelial cells were induced for 24h to get the endoplasmic reticulum stress-induced apoptosis model.

### Determination of the level of mRNA and protein and apoptosis rates in JNK, CHOP and Caspase-12

The expression levels of mRNA in JNK, CHOP and Caspase-12 mRNA which were interfered before and after in two groups of dogs were measured by real-time fluorescence quantitative PCR(ABI7500 Fast, Sangon, China), and the protein in the same situation was measured by Western Blot (Thermo SCIENTIFIC, Thermo Fisher Scientific, USA). The rear suspension cells were washed twice after centrifugation to make the cell density reach 1×106/m, and then 5μl Annexin V-PE and 5μl 7-ADD were added for staining. The Flow cytometry (BD, USA) was used to detect the apoptosis rate of JNK, CHOP and Caspase -12 before and after RNA interference in two groups.

### Analysis of histological

All animals were sacrificed by the way of intravenous potassium chloride, and then all specimens (Liver, Lung and Kidney) were collected (after T_5_ point), fixed routinely in 10% formalin solution and embedded in paraffin. Hematoxylin-eosin-stained sections were prepared to observe the pathological tissues under the light microscope.

### Sample preparation for the metabonomic analysis

The sera were thawed at 4 °C. 100μl serum was deproteinized with 2 volumes of acetonitrile, followed by centrifugation at 13000g for 20min, and the supernatant was dried in the vacuum centrifuge and stored at -20 °C. The samples were re-dissolved in 100μl water and acetonitrile (2:8) for further analysis. The quality control samples were prepared by mixing 10μl of each serum sample, and the samples were treated with the same way. All the samples were analyzed by UPLC (Waters Corp, Milford, USA) coupled to Triple Q TOPF-MS(AB SCIEX, Framingham, USA), which were operated in both the positive and negative ion mode (full scan mode from m/z 80-1000). A 2.1mm×100mm, ACQUITYTM 1.7μm C8 column (Waters, Ireland) had been used for the positive ion mode. The mobile phase contained (A) water with 0.1% formic acid and (B) acetonitrile. From the start to 0.5min, buffer B was kept at 5% and linearly increased to 100% in 24min, kept for 4 minutes and reduced to 5% in 0.1min. A 2.1mm×100mm, ACQUITYTM HSS 1.8mm T_3_ column (Waters Ireland) had been used for the negative ion mode. The mobile phase contained (C) 6.5mM NH_4_HCO_3_ in water and (D) 6.5 mM NH_4_HCO_3_ in 95% methanol/water. From the start to 1 min, buffer D was kept at 2% and linearly increased to 100% in 18 min, and then kept for 3 min. Finally, buffer D was reduced to 2%, and the column temperature was kept constant at 50 °C. About the mass spectrometry, capillary voltage was set as 3100V in the positive mode while 2500V in the negative mode.

### Data analysis and statistical processing

Statistical comparisons were performed using the SPSS software (version 19.0). The Ion features of Triple Q TOF were arranged by Markerview workstation (AB SCIEX, USA). The principal component analysis (PCA) was performed by the SIMCA-P software (version11.0, Umetrics, Umea, Sweden). Hot map was obtained by MeV version 4.5.1software.

Data were expressed as mean ± SD. The difference between means with a single variable was analyzed by independent sample t-test. A repeated measurement analysis of variance test with a post hoc LSD pair-wise comparison was performed in the process with the progress in treatment. The P <0.05 was considered statistically significant for all analyses.
